# Interactome of PTH-Regulated miRNAs and Their Predicted Target Genes for Investigating the Epigenetic Effects of PTH (1–34) in Bone Metabolism

**DOI:** 10.3390/genes13081443

**Published:** 2022-08-13

**Authors:** Lucija Ana Vrščaj, Janja Marc, Barbara Ostanek

**Affiliations:** Department of Clinical Biochemistry, Faculty of Pharmacy, University of Ljubljana, 1000 Ljubljana, Slovenia

**Keywords:** osteoporosis, osteoporosis treatment, microRNA, parathyroid hormone, teriparatide, network, Cytoscape, biological pathway, gene enrichment biomarker

## Abstract

Osteoporosis is a metabolic bone disease that mostly affects the elderly. A lot of drugs are available, mostly with an antiresorptive effect but just a few with an osteoanabolic effect, meaning they promote bone building. PTH (1-34) or teriparatide is an osteoanabolic drug, but its efficacy varies between individuals. We performed a literature review and extracted a dataset of 62 microRNAs (miRNAs) from 10 different studies; predicted miRNA target interactions (MTIs) were obtained with the help of four software tools: DIANA, miRWalk, miRDB and TargetScan. With the construction of an interactome of PTH-regulated miRNAs and their predicted target genes, we elucidated miR-146a-5p, miR-551b-5p, miR-205-3p, miR-33a-3p, miR-338-5p as miRNAs with the most interactions and miR-410-3p as the miRNA targeting bone-related pathways with the highest significance. These miRNAs could help in further understanding the mechanism of action of PTH on bone metabolism and osteoporosis. They also have the potential for novel network-based biomarkers for osteoporosis treatment efficacy and safety and as new therapeutic targets.

## 1. Introduction

Osteoporosis is the most common metabolic bone disease in older women, and its incidence increases with age in both sexes. Osteoporosis is characterized by low bone mass and deteriorated bone microarchitecture due to an imbalance between bone resorption by osteoclasts and bone formation by osteoblasts resulting in increased bone fragility [1,2]. With age, differentiation of mesenchymal stem cells (MSCs) in the bone marrow shifts from osteogenesis to adipogenesis, therefore, adipocytes replace osteoblasts, and bone regeneration is decreased [3]. 

Osteoporosis is one of the main health problems in developed societies, as osteoporotic fractures are extremely common, significantly impair the quality of life of patients who suffer from them, increase patient mortality and cause huge treatment costs [1]. It is estimated that there are 200 million people in the world with osteoporosis, and due to the general aging of the world’s population, this number will increase in the coming years [4]. It is more common in women, and every third 50-year-old will suffer at least one osteoporotic fracture in their lifetime. The most common are vertebral, hip and wrist fractures [5]. 

There are several medications available that can reduce the risk of fracture. We classify them into two groups, namely antiresorptives and osteoanabolics. Antiresorptives are the most commonly used but are less effective than osteoanabolics [6]. Additional efforts were therefore devoted to drugs that would promote bone formation, and the first osteoanabolic drug was teriparatide [7]. Teriparatide is a recombinant peptide identical to the sequence of the first 34 amino acids of endogenous human parathyroid hormone (PTH). Studies show that PTH reduces osteoblast apoptosis and activates quiescent bone-lining cells [8]. It has also been shown to inhibit the differentiation of MSCs into adipocytes and stimulate their differentiation into osteoblasts [9]. However, PTH also has catabolic effects on the bone, as shown in previous studies [10,11]. For more successful approaches to anabolic treatment, it is important to have a good knowledge of signaling pathways and various mechanisms that regulate the functioning of bone cells and the entire process of bone regeneration. Even though a lot of progress was made, the exact mechanism of teriparatide action on bone development and homeostasis is still not fully understood. 

In our research, we were focused on the mechanisms of teriparatide action on bone through microRNAs (miRNAs). These noncoding RNAs are epigenetic modifiers that regulate target gene expression in two ways. Either by inhibiting translation and protein synthesis or by promoting mRNA degradation [12]. Existing work in the literature has shown that PTH affects at least 62 miRNAs, which we present in this study, but there is still a lack of concrete evidence as most of these studies are performed in vitro. These studies were performed on different cell types, e.g., human osteoblasts [13], rat osteoblasts and human MSCs [14], but to date, only one study of selected miRNAs has been performed in patients in relation to the efficacy of teriparatide treatment [15]. 

**Figure 1:**
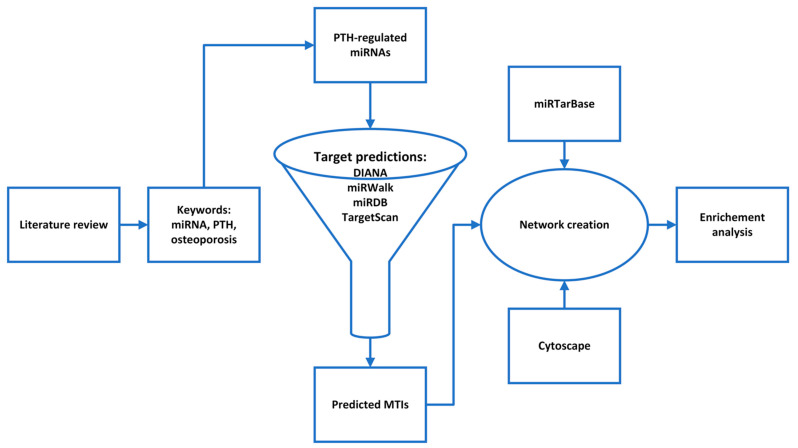
A flowchart of the study design.

The aim of this study was to obtain new insights into the teriparatide action on bone by an in silico analysis of the PTH-regulated miRNAs. First we selected PTH-regulated miRNAs by a literature review and then we constructed their interactome. To do so, the predicted target genes of all PTH-regulated miRNAs were grouped into a single interactome, and potentially relevant combinations of miRNA and target genes/proteins were identified. With a pathway enrichment analysis, we found the most important signaling pathways that play a role in PTH-regulated miRNA action on bone (Figure 1). The generation of such an interactome provides a basis for a better understanding of PTH action on bone cells and the identification of new markers of teriparatide treatment efficacy and safety or new therapeutic strategies for postmenopausal osteoporosis.

## 2. Materials and Methods

### 2.1. Literature Review and miRNA Selection

The purpose of the study was to identify the PTH-regulated miRNAs in the treatment of osteoporosis with teriparatide. We performed a systematic review of the literature. Using the PICO approach (P—population, I—intervention, C—comparison, O—outcome), we put together a research question: “Does teriparatide or PTH treatment influence miRNAs?” We selected the keywords: PTH, teriparatide and miRNA. With these, we conducted a literature search in three online databases: Web of Science, Science Direct and the MEDLINE database, based on the PRISMA approach [16]. The MEDLINE bibliographic database was accessed via the PubMed electronic library (https://pubmed.ncbi.nlm.nih.gov/) (accessed on 18 February 2022). We used previously selected keywords with relational and logical operators in the search: (miRNA) AND (PTH OR teriparatide). The inclusion criteria were osteoporosis and serum, bone or bone cells, exclusion criteria were chondrocytes, secondary osteoporosis, no treatment with PTH or PTH (1-34), no direct effects of PTH on miRNA expression.

We used the miRNA Tissue Expression Database (miTED) (https://dianalab.e-ce.uth.gr/mited/#/) (accessed on 2 August 2022) to check whether all miRNAs are expressed in bone [17].

### 2.2. Target Predictions and Enrichment Analysis

We used four bioinformatic tools for miRNA-target predictions:

- DIANA-microT web server v5.0 (http://diana.imis.athena-innovation.gr/DianaTools/index.php) (accessed on 10 March 2022), where target prediction is performed by a DIANA-microT-CDS prediction algorithm, which is the only algorithm that also searches for matches in 5’UTR [18].
- miRWalk v 2.0 (http://mirwalk.umm.uni-heidelberg.de/) (accessed on 12 March 2022), where target prediction is conducted with a machine learning algorithm [19].
- miRDB (http://mirdb.org.) (accessed on 13 March 2022), where target prediction is conducted by MirTarget, a machine learning algorithm [20].
- TargetScanHuman v 8.0 (https://www.targetscan.org/vert_80/) (accessed on 15 March 2022), where target prediction is conducted by a TargetScan algorithm, which matches miRNA seed regions with 8mer, 7mer and 6mer sites in 3’UTR [21].

As these different tools use different algorithms for target predictions and the interactions are then ranked in different ways, we compared the interactions between tools and extracted the interactions that appeared in at least 3 tools.

We used miRTarBase v8.0 (https://mirtarbase.cuhk.edu.cn/~miRTarBase/miRTarBase_2022/php/index.php) (accessed on 17 March 2022) for exploring validated miRNA-target interactions (MTIs). The validation is divided into strong experimental evidence, where a reporter assay or Western blot is used, and weak experimental evidence, where microarray or pSILAC is used. We only selected MTIs that are supported by strong experimental evidence [22]. We need to note that the validated interactions in miRTarBase had to be searched manually, as osteoporosis is not a disease that is indexed in the database. That may be due to the small set of validated interactions, associated with osteoporosis. Another problem is that miRTarBase is manually updated, so some validated interactions could be found in the literature but not in the database. That is probably also due to the rapid pace of new studies and developments in this field; therefore, it may be challenging to adequately curate such a large database.

Networks were created with the Cytoscape tool (https://cytoscape.org) (accessed on 30 March 2022), which was also used for analyzing the created networks [23]. PTH-regulated miRNAs were then analyzed for enrichment in biological pathways using miRPath v.3 (http://diana.imis.athena-innovation.gr/DianaTools/index.php) (accessed on 15 April 2022), which uses the microT-CDS algorithm to predict the dataset miRNAs’ target genes and identify biological pathways in which they are enriched. This was performed with the KEGG analysis tool, where a *p*-value threshold of 0.05 was used [24]. Furthermore, a heatmap of all the significantly targeted pathways by the selected miRNAs was created using miRPath. We used a Fisher’s exact test with an FDR correction and *p*-value threshold of 0.05.

## 3. Results

### 3.1. Selection of Human miRNAs Influenced by PTH

**Figure 2:**
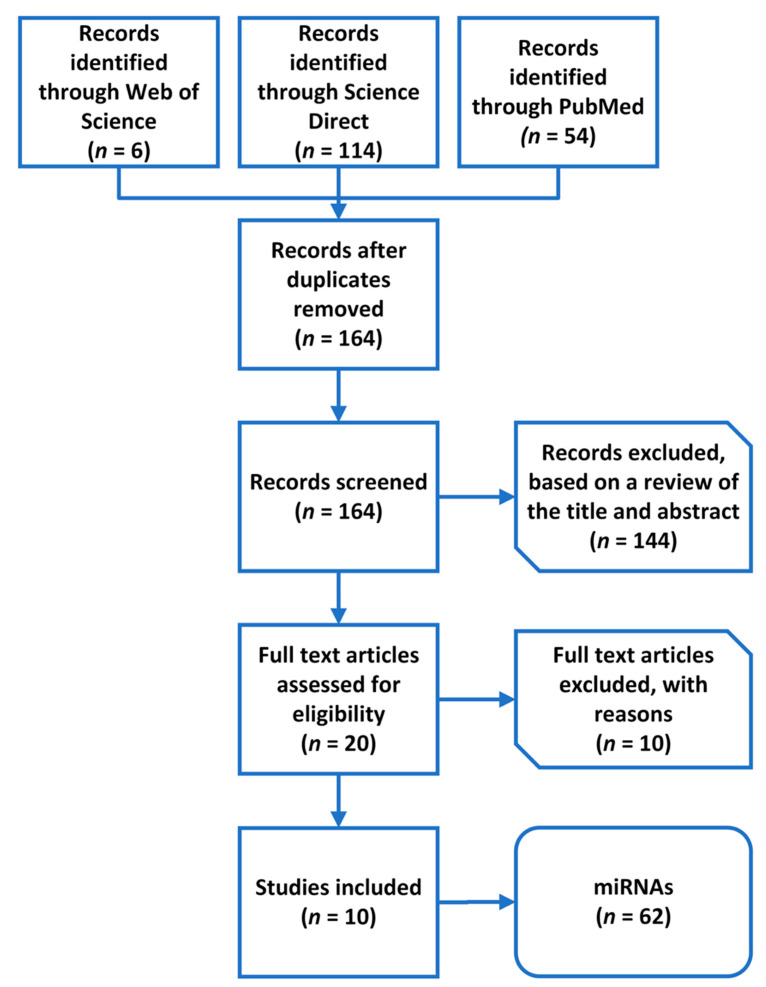
A flowchart of the literature review process and microRNA (miRNA) selection.

In the first step of the literature review, our search returned 174 results. A total of 164 results remained after excluding duplicates. In the first phase, the articles were excluded based on a review of the title and summary. Thus, we eliminated 144 records because they described other diseases or conditions. We continued to screen the hits according to the strength of the evidence; therefore, we eliminated reviews and articles that did not answer our research question (*n* = 7). In the end, we included 10 studies (Figure 2). 

After removing duplicates, a total of 62 miRNAs associated with PTH were extracted from the 10 selected studies (Table 1 and Table 2).

Table 1 summarizes the articles relevant to our study. The studies are quite different; five studies are in vitro, four are in vivo, and one is a combination of both. Only two studies included teriparatide treatment in humans, and other studies were either in animal models or in cell lines or primary cells. The aims of the studies also vary, but we were able to select ones where PTH directly affects miRNAs. Anastasilakis et al. also included the miRNAs that were related to bone mineral density and bone turnover markers, but the expression of these miRNAs was not significantly different in teriparatide treatment versus control; therefore, we excluded those particular miRNAs from further analysis. We continued with the selection of miRNAs directly affected by PTH (Table 2). We excluded miRNAs exclusively expressed in rats and included only miRNAs that are conserved through different species.

Only five of the miRNAs in Table 2 were not examined in bone tissue or bone cells but in serum. Therefore, we wanted to confirm that they are also expressed in bone. We used the miTED database and confirmed that miR-23a-3p, miR-33a-3p, miR-133a-3p, miR-29a-3p, miR-338-3p are also expressed in bone tissue.

### 3.2. MTI Predictions

We used four different bioinformatic tools for MTI predictions and put together all these interactions to create a network using the Cytoscape tool.

We also used the Cytoscape tool to analyze the created network. As the dataset was large, the miRNAs had 39,111 interactions, e.g., miR-146a-5p had 7493 interactions, and nine miRNAs had more than a thousand interactions. The most prominent miRNAs were miR-146a-5p, miR-551b-5p, miR-205-3p, miR-33a-3p and miR-338-5p. The interactome of these miRNAs together with miR-410-3p, which was the hit in the following heat map analysis (3.4), is presented in Supplement S1. *DGKH* and *TNRC6B* were the target genes of the most miRNAs with 32 and 29 interactions, respectively, and were followed by *ZNF704*, *INO80D* and *NFAT5*, which were the target of 28, 24 and 24 miRNAs, respectively. We were not able to identify any smaller subnetworks. Therefore, we continued with a KEGG pathway enrichment analysis.

### 3.3. KEGG Pathway Enrichment Analysis

We performed a KEGG pathway enrichment analysis on all miRNAs and the predicted genes with the miRPath 3.0 tool. Our dataset of 62 miRNAs were enriched in 58 pathways which are displayed in Table 3.

### 3.4. Heatmap of Pathways Union Enrichment Analysis

After the initial enrichment analysis, we also created a heatmap of a pathway’s union with all the significantly targeted pathways. The results are shown in Supplement S2. miR-132-3p and miR-212-3p significantly target the highest number of pathways, namely eight. Of these, the most significant target is the TGF-beta signaling pathway. The prion diseases pathway is targeted with the highest significance by six miRNAs, namely miR-30d-3p, miR-205-3p, miR-130b-3p, miR-301a-3p, hsa-301b-3p and miR-410-3p. miR-410-3p, on the other hand, is the one targeting the most significant bone-related pathways: TGF-beta signaling pathway, signaling pathways regulating the pluripotency of stem cells and the Hippo signaling pathway but also targets proteoglycans in cancer. Furthermore, miR-551b-5p preferentially targets bone-related pathways: Hippo, TGF-beta, FoxO signaling pathways, signaling pathways regulating the pluripotency of stem cells, while it also targets morphine addiction. Both 410-3p and miR-551b-5p target non-bone-related pathways with lower significance. Only miR-33a-3p miRNA significantly targets more than one bone-related pathway, namely three (TGF-beta, estrogen signaling pathway and signaling pathways regulating pluripotency of stem cells), while not targeting any other pathways. There were two miRNAs, miR-18a-5p and miR-338-5p, that significantly targeted only one bone-related pathway, the Hippo signaling pathway and TGF-beta signaling pathway, respectively. All of these miRNAs could help further explain the complex mechanism of the PTH effect on bone health and osteoporosis.

## 4. Discussion

Our in silico analysis showed that PTH (1-34) effects on genes are most probably mediated by miR-146a-5p, miR-551b-5p, miR-205-3p, miR-33a-3p, miR-338-5p and miR-410-3p, and genes with the highest number of miRNAs were *DGKH*, *TNRC6B*, *ZNF704*, *INO80D* and *NFAT5*. We performed a detailed literature review, selected relevant miRNAs, predicted their target genes with four software tools, created a large network of MTIs and performed an enrichment analysis with a heatmap.

In the present study, we first formed a large network of predicted MTIs, based on data of PTH-regulated miRNAs, selected with a detailed literature review. Relevant studies were obtained using inclusion criteria: osteoporosis and serum, bone or bone cells and exclusion criteria: chondrocytes, secondary osteoporosis, no treatment with PTH or no direct effects of PTH on miRNA expression. After the selection process, we included 10 studies and 62 miRNAs in our study. The network that we created is the first network of PTH-regulated miRNAs and their predicted target genes. This network did not contain any subnetworks and contained 39,111 interactions. The nodes with the most interactions were most of the miRNAs with miR-146a-5p, miR-551b-5p, miR-205-3p, miR-33a-3p and miR-338-5p at the top. Target genes with the highest number of miRNAs were *DGKH*, *TNRC6B*, *ZNF704*, *INO80D* and *NFAT5*, but according to the literature, none of these were ever directly associated with PTH or osteoporosis. Although, we can note that *NFAT5,* which encodes the nuclear factor of activated T cells 5, exhibits some osteoprotective properties via interacting with osteoprotegerin (OPG). *NFAT5* encodes a transcription factor that binds to the *OPG* promoter region and upregulates its expression [33]. OPG, as a decoy receptor, inhibits RANKL effects on the development and activation of osteoclasts and protects against excessive bone resorption [34]. A noteworthy gene is also *ZBTB20*, which encodes the zinc finger and BTB domain containing 20 and is the predicted target of the top 10 miRNAs that target multiple genes. It is worth mentioning that *ZBTB20* is involved in the endochondral ossification through repression of *SOX9* transcription and thus regulating the terminal differentiation of hypertrophic chondrocytes [35]. Endochondral ossification is an important part of embryological skeletal development and also of fracture healing later in life, in which cartilage is replaced by bone [36,37]. Downregulation of *ZBTB20* in chondrocytes slows down endochondral ossification [35], which is known to be impaired in osteoporosis [38]. 

To identify already experimentally validated interactions in our dataset, we searched the miRTarBase database and chose only those verified by methods deemed as strong experimental evidence. These include Western blot and reporter assay, as opposed to microarrays, next-generation sequencing (NGS) and pSILAC, which are weak experimental evidence. Right now, the consensus about the strength of the evidence when validating MTIs has not been achieved. The red-colored edges in Supplement S1 show strong validation, but an expansion may be needed in the future if the consensus changes. Out of the top five genes, two interactions were validated, namely *NFAT5*-miR-31-5p and *NFAT5*-miR-146a-5p. Furthermore, Weigl et al. showed that PTH downregulates miR-31-5p, and consequently, NFAT5 is predicted to be upregulated, leading to more osteoprotective properties [25,33]. In the case of miR-146-5p, it is not so simple, as PTH upregulates it after 4 and 8 h of exposure or downregulates it after 1 and 2 h of exposure in vitro [29].

In the next step, we conducted an enrichment analysis for PTH-regulated miRNAs to elucidate the mechanisms of PTH-regulated miRNAs action on pathways that are known to play a role in bone biology and osteoporosis. The enrichment analysis provided 58 pathways, indicating that PTH-regulated miRNAs are involved in a variety of biological pathways, including those important in bone biology, namely Hippo, WNT, AMPK, FoxO, PI3K-Akt, TGF-beta signaling pathways and other signaling pathways regulating the pluripotency of stem cells [39,40,41,42,43,44,45]. Subsequently, we created a heatmap of a pathway’s union of all the significantly targeted pathways by the miRNAs in our dataset to obtain further insight into the dataset’s PTH-regulated miRNAs involvement in signaling pathways. 

According to the heatmap analysis, miR-132-3p and miR-212-3p target the highest number of pathways, of which the TGF-beta signaling pathway was the most significant. Both miRNAs are known to inhibit osteogenic differentiation of human bMSCs [46,47], and in the TGF-beta pathway, they target *SMAD2* and *SMAD5*. The interactions between miR-132-3p, miR-212-3p and *SMAD2* have been validated. [44,48,49,50]. The *SMAD2* gene encodes the SMAD family member 2 (SMAD2) protein, which is a specific mediator of the TGF-beta signaling pathway [44]. SMAD family members transmit signals from all receptors activated by the TGF-beta superfamily members to target genes in the nucleus [37,51]. SMAD5 is a transcription factor activated by BMP2 receptors. Forming a complex with SMAD4, it translocates into the nucleus to activate RUNX2 [52], which is associated with PTH and osteoporosis [53]. Kocijan et al. showed in an in vivo model that miR-132-3p and miR-212-3p are the most upregulated miRNAs by PTH in bone [27]. These two miRNAs also target other pathways; of these, the Hippo signaling pathway and signaling pathways regulating the pluripotency of stem cells are most similar to the TGF-beta signaling pathway. Other pathways are not bone-related, and both miRNAs have been shown to play a role in cancer—miR-132-3p is associated with colorectal cancer [54], and miR-212-3p is associated with hepatocellular carcinoma [55]. miR-410-3p stands out in the heatmap analysis as the one that most significantly targets bone-related pathways. It very significantly targets the TGF-beta signaling pathway, signaling pathways regulating the pluripotency of stem cells and the Hippo signaling pathway, but also targets proteoglycans in cancer. It is upregulated by PTH, but no previously published studies showed any connections to bone biology. miR-146a-5p from our initial interactome analysis is the most involved in Hippo, WNT and TGF-beta pathways but targets none of them with high significance. It is also the only miRNA that has validated interactions with target genes in these three pathways. Interactions between miR-146a-5p and *CCND2*, *NFAT5*, *SMAD2* and *SIRT1* are validated. Of these, *NFAT5* and *SIRT1* have a direct effect on bone remodeling [33,56,57,58]. The other miRNAs have not yet been connected to these bone-related pathways.

miRNAs listed above with the highest number of interactions in the interactome and those with the highest significance in bone-related pathways in the enrichment analysis and their targets represent the most interesting candidates for further study of their involvement in the PTH epigenetic mechanism of action. After extensive experimental validation, they could serve as new potential biomarkers of teriparatide treatment efficacy or new therapeutic targets in osteoporosis. Evidence for such opportunities already exists for some of the top miRNAs. 

miR-146a-5p, from the initial interactome, targets most of the significant genes in all bone-related pathways, which means that it could be a very important miRNA in the process of bone homeostasis, osteoporosis and osteoporosis treatment with PTH, even though it does not significantly target any bone-related pathways. Previously published data, however, confirm its importance in bone health, as it was shown in a mice knockout model in vivo that miR-146a-5p regulates bone mass via *SIRT1* [59], and miR-146a-5p could be considered a therapeutic option for osteoporosis. 

miRNAs have been well studied for their potential use as osteoporosis biomarkers in previous studies [60,61,62,63], but in the case of the treatment of osteoporosis with PTH, only two studies have been conducted in terms of determining a potential circulating biomarker [15,26]. Of these, only Anastasilakis et al. studied the correlation between the changes in miRNA expression and teriparatide treatment efficacy, namely BMD and bone turnover markers (BTMs) [15]. They showed that teriparatide decreases the relative expression of miR-133a-3p after 12 months of treatment and miR-33-3p after 3 months. The former miRNA has already been proposed as a biomarker for postmenopausal osteoporosis, and the latter miRNA is the miRNA with one of the highest number of gene interactions in our dataset. As a biomarker for predicting treatment efficacy, none of these miRNAs were significant, but the relative expression of miR-124-3p at 3 months could predict BMD at 12 months [15]. Using this miRNA, we could better predict the treatment efficacy of PTH and adjust the treatment accordingly, but more studies need to confirm these results. Moreover, the miRNAs studied could be expanded to include those found to be relevant in our analysis since Anastasilakis et al. included 16 miRNAs previously known to be involved in bone metabolism and not those specifically regulated by PTH.

The current study has some limitations. First, the studies included are quite different. Some of the studies were in vitro, some were in vivo, and of these, only two were in humans. In vitro studies outweighed in vivo studies five to four. The studies also used different methodologies for miRNA selection: NGS, microarrays, and literature review. Second, the studies focused on miRNAs from different tissues. Third, some MTI databases could not recognize all the miRNAs, and we excluded them from the study.

In the present study, we performed a literature review and extracted a dataset of 62 miRNAs from 10 different studies; predicted MTIs were obtained with the help of four software tools. All data were visualized in the form of networks, and a network analysis revealed important hub genes and miRNAs, which have the potential for novel network-based biomarkers for osteoporosis treatment efficacy and adverse drug effects. Our analysis revealed miR-146a-5p, miR-551b-5p, miR-205-3p, miR-33a-3p, miR-338-5p and miR-410-3p as PTH-regulated miRNAs, which were also enriched in pathways known to play a part in bone biology and osteoporosis (Supplement S1). MTIs and biological pathways associated with osteoporosis could help in further understanding the mechanism of action of PTH on bone development and osteoporosis. After experimental validation, these miRNAs could serve as new potential biomarkers of teriparatide treatment efficacy or new therapeutic targets in osteoporosis. Future tools for clinicians using well-scored circulating miRNAs or their combinations with other types of circulating bone biomarkers could contribute to better osteoporosis management. Similar network-based approaches could also be extended to miRNAs differentially expressed between osteoporotic patients and controls or with other treatments for the identification of new diagnostic markers and new osteoporosis drugs.

## Figures and Tables

**Table 1:** The final set and characteristics of included studies obtained by a detailed literature review.

Author	Year	Type of Study	miRNAs	Drug	Tissue of miRNA Extraction	Aim of Study
Weigl M, et al. [25]	2021	in vivo (A-rats)	miR-203b-3p	Teriparatide	Serum and bone	Evaluate the time-dependent changes of circulating miRNAs in serum of ovariectomized rats during zoledronic acid and teriparatide treatment; assessment of in vivo and ex vivo longitudinal changes in bone microstructure; association of miRNAs to bone structure parameters
			miR-31-5p			
			miR-378a-5p			
			miR-188-5p			
			miR-375-3p			
			miR-107			
			miR-183-5p			
			miR-203a-3p			
			miR-30d-3p			
			miR-34a-5p			
Yavropoulou MP, et al. [26]	2020	in vivo (H-women)	miR-23a-3p	Teriparatide	Serum	Evaluate the effect of sequential treatment with denosumab following zoledronate or teriparatide treatment on miRNA expression in postmenopausal women with osteoporosis
			miR-29a-3p			
			miR-338-3p			
			miR-21a-5p			
Kocijan R, et al. [27]	2020	in vivo (A-rats)	miR-212-5p	Teriparatide	Serum and bone	Evaluate the expression of bone-related miRNAs in bone and serum of ovariectomized rats during treatment with zoledronic acid and teriparatide
			miR-125b-1-3p			
			miR-10b-5p			
			miR-3473			
			miR-125a-3p			
			miR-196a-5p			
			miR-183-5p			
			miR-212-3p			
			miR-132-5p			
			miR-132-3p			
			miR-455-3p			
			miR-433-3p			
			miR-182-3p			
			miR-151a-3p			
			miR-320b			
			miR-17-5p			
			miR-18a-5p			
			miR-19a-3p			
			miR-20b-5p			
			miR-32-3p			
			miR-106b-5p			
			miR-130b-3p			
			miR-130b-5p			
			miR-203a-3p			
			miR-301a-3p			
			miR-301b-3p			
			miR-363-3p			
Anastasilakis AD, et al. [15]	2018	in vivo (H-women)	miR-33a-3p	Teriparatide	Serum	Evaluate the differential expression of miRNAs linked to bone metabolism in low bone mineral density postmenopausal women when treated with teriparatide or denosumab
			miR-133a-5p			
Akshaya N, et al. [28]	2021	in vitro (C-rat osteoblasts)	miR-338-5p	Rat PTH (1-34)	Osteoblasts	Identify and characterize miRNAs that target Runx2 in the PTH-stimulation of MMP-13 expression in rat osteoblastic cells
			miR-384			
			miR-325			
			miR-6333			
			miR-290			
Malavkia D, et al. [29]	2020	in vitro (C-rat osteoblasts UMR 106-01 cell line)	miR-551b-5p	Rat PTH (1-34)	Osteoblasts	Identify and validate the functional roles of miRNAs that target HDAC4 to affect MMP-13 expression in rat osteoblasts
			miR-186-5p			
			miR-221-3p			
			miR-873-3p			
			miR-132-5p			
			miR-187-5p			
			miR-18a-3p			
			miR-146a-5p			
			miR-146b-5p			
			miR-143-3p			
			miR-139-3p			
Arumugam B, et al. [30]	2019	in vitro (C-human bone marrow mesenchymal stem cells)	miR-6797-5p	PTH (1-34)	Bone marrow MSCs	Identify PTH-induced stimulation of Runx2, lncRNAs and miR-6797-5p in human marrow stromal cells
Karvande A, et al. [31]	2018	in vivo (A-mice) + in vitro (C-mice osteoblasts)	miR-451a	PTH (1-34)	Osteoblasts	Evaluate PTH effects on glucose-dependent miR-451a in mice
Mohanakrishnan V, et al. [32]	2018	in vitro (C-rat osteoblasts UMR 106-01 cell line)	miR-532-5p	Rat PTH (1-34)	Osteoblasts	Evaluate PTH effects on miRNAs that target MMP-13
			miR-511-5p			
			miR-141-3p			
			miR-410-3p			
			miR-346			
			miR-494-3p			
			miR-3580-5p			
Laxman N, et al. [13]	2016	in vitro (C-human osteoblasts)	miR-30c-5p	Teriparatide	Osteoblasts	Evaluate changes in miRNA levels in human osteoblasts after treatment with teriparatide or denosumab
			miR-203a-3p			
			miR-203b-3p			
			miR-205-3p			
			miR-320b			

Legend: C-cells, A-animals, H-humans.

**Table 2:** MicroRNAs (miRNAs) influenced by PTH.

miRNA	Effect of PTH on miRNA Expression	Comments	Reference
miR-212-5p	↑		Kocijan et al. [27]
miR-125b-1-3p	↑		Kocijan et al. [27]
miR-10b-5p	↑		Kocijan et al. [27]
miR-125a-3p	↑		Kocijan et al. [27]
miR-196a-5p	↑		Kocijan et al. [27]
miR-183-5p	↑/↓	[27] report an increase in expression, [25] report a decrease in expression	Kocijan et al. [27], Weigl et al. [25]
miR-212-3p	↑		Kocijan et al. [27]
miR-132-5p	↑		Kocijan et al. [27], Malavkia et al. [29]
miR-132-3p	↑		Kocijan et al. [27]
miR-455-3p	↑		Kocijan et al. [27]
miR-433-3p	↑		Kocijan et al. [27]
miR-182-3p	↑		Kocijan et al. [27]
miR-151a-3p	↑		Kocijan et al. [27]
miR-320b	↑/↓	[27] report an increase in expression, [13] report a decrease in expression between two time points, namely 2 and 24 h after teriparatide application	Kocijan et al. [27], Laxman et al. [13]
miR-17-5p	↓		Kocijan et al. [27]
miR-18a-5p	↓		Kocijan et al. [27]
miR-19a-3p	↓		Kocijan et al. [27]
miR-20b-5p	↓		Kocijan et al. [27]
miR-32-3p	↓		Kocijan et al. [27]
miR-106b-5p	↓		Kocijan et al. [27]
miR-130b-3p	↓		Kocijan et al. [27]
miR-130b-5p	↓		Kocijan et al. [27]
miR-203a-3p	↓		Kocijan et al. [27], Weigl et al. [25]
miR-301a-3p	↓		Kocijan et al. [27]
miR-301b-3p	↓		Kocijan et al. [27]
miR-363-3p	↓		Kocijan et al. [27]
miR-203b-3p	↓		Weigl et al. [25], Laxman et al. [13]
miR-31-5p	↓		Weigl et al. [25]
miR-378a-5p	↓		Weigl et al. [25]
miR-188-5p	↓		Weigl et al. [25]
miR-107	↓		Weigl et al. [25]
miR-30d-3p	↓		Weigl et al. [25]
miR-34a-5p	↓		Weigl et al. [25]
miR-375-3p	↓		Weigl et al. [25],
miR-23a-3p	↑		Yavropoulou et al. [26]
miR-33a-3p	↓		Anastasilakis et al. [15]
miR-133a-3p	↓		Anastasilakis et al. [15]
miR-29a-3p	↑		Yavropoulou et al. [26]
miR-338-3p	↑		Yavropoulou et al. [26]
miR-551b-5p	↑	Increased expression 1, 2, 4 and 8 h after PTH application	Malavkia et al. [29]
miR-186-5p	↑		Malavkia et al. [29]
miR-221-3p	↑		Malavkia et al. [29]
miR-873-3p	↑	Increased expression 2 and 8 h after PTH application	Malavkia et al. [29]
miR-187-5p	↑		Malavkia et al. [29]
miR-18a-3p	↑		Malavkia et al. [29]
miR-146a-5p	↑/↓ *	Decreased expression 1 and 2 h after PTH application, increased expression 4 and 8 h after PTH application	Malavkia et al. [29]
miR-146b-5p	↑/↓ *		Malavkia et al. [29]
miR-143-3p	↑/↓ *	Increased expression 1, 2 and 8 h after PTH application, decreased expression 4 h after PTH application	Malavkia et al. [29]
miR-139-3p	↑/↓ *	Increased expression 1, 2 and 4 h after PTH application, decreased expression 8 h after PTH application	Malavkia et al. [29]
miR-451a	↑		Karvande et al. [31]
miR-6797-5p	↑		Arumugam et al. [30]
miR-532-5p	↓		Mohanakrishnan et al. [32]
miR-511-5p	↓		Mohanakrishnan et al. [32]
miR-141-3p	↑		Mohanakrishnan et al. [32]
miR-410-3p	↑		Mohanakrishnan et al. [32]
miR-346	↑		Mohanakrishnan et al. [32]
miR-494-3p	↑		Mohanakrishnan et al. [32]
miR-30c-5p	↓		Laxman et al. [13]
miR-205-3p	↓		Laxman et al. [13]
miR-338-5p	↑/↓ *	Decreased expression 1 and 2 h after PTH application; increased expression 8 h after PTH application	Akshaya et al. [28]
miR-384-5p	↑/↓ *		Akshaya et al. [28]
miR-325-3p	↑/↓ *	Decreased expression 1 and 2 h after PTH application; increased expression 4 and 12 h after PTH application	Akshaya et al. [28]

Legend: ↑—PTH increases miRNA expression, ↓—PTH decreases miRNA expression, ↑/↓—PTH increases or decreases miRNA expression in different studies, *—the effects of PTH on miRNA expression differentiate through the study.

**Table 3:** Enriched pathways from the dataset. A total of 62 miRNAs were enriched in 58 pathways.

KEGG Pathway	*p*-Value	#Genes	#miRNAs
Proteoglycans in cancer	6.32 × 10^−16^	150	57
Mucin type O-Glycan biosynthesis	1.33 × 10^−12^	24	31
Axon guidance	1.15 × 10^−9^	97	54
Renal cell carcinoma	2.60 × 10^−8^	58	55
Adherens junction	7.54 × 10^−7^	61	50
Signaling pathways regulating pluripotency of stem cells	1.61 × 10^−6^	105	53
Pathways in cancer	1.61 × 10^−6^	270	58
Hippo signaling pathway	4.02 × 10^−6^	109	50
Ras signaling pathway	4.02 × 10^−6^	154	57
Wnt signaling pathway	6.65 × 10^−6^	105	54
ECM-receptor interaction	9.06 × 10^−6^	54	44
Fatty acid biosynthesis	1.47 × 10^−5^	8	17
ErbB signaling pathway	1.69 × 10^−5^	67	56
Rap1 signaling pathway	2.77 × 10^−5^	148	55
Circadian rhythm	4.84 × 10^−5^	28	42
Focal adhesion	5.37 × 10^−5^	146	56
AMPK signaling pathway	8.27 × 10^−5^	90	55
Estrogen signaling pathway	0.000105	69	55
Endocytosis	0.000108	144	57
Glioma	0.000151	48	51
N-Glycan biosynthesis	0.000247	34	34
Pancreatic cancer	0.000247	50	51
GABAergic synapse	0.000248	60	51
MAPK signaling pathway	0.000347	170	56
FoxO signaling pathway	0.000364	98	54
Thyroid hormone signaling pathway	0.000472	84	50
Prostate cancer	0.000495	66	51
TGF-beta signaling pathway	0.000533	59	48
PI3K-Akt signaling pathway	0.000533	222	59
Gap junction	0.000679	62	52
Glycosaminoglycan biosynthesis—keratan sulfate	0.001220	13	22
Oxytocin signaling pathway	0.001220	109	54
Melanoma	0.001279	55	48
Lysine degradation	0.001592	34	49
Prion diseases	0.001678	18	32
Morphine addiction	0.001678	64	52
Prolactin signaling pathway	0.001678	50	53
Non-small cell lung cancer	0.001678	41	53
Cell cycle	0.002452	84	51
Ubiquitin mediated proteolysis	0.002452	94	54
Glutamatergic synapse	0.002703	77	54
Choline metabolism in cancer	0.003314	73	56
Insulin signaling pathway	0.005820	96	56
Regulation of actin cytoskeleton	0.006559	144	57
Melanogenesis	0.007311	69	48
Colorectal cancer	0.007832	46	48
Endometrial cancer	0.008261	39	51
Hedgehog signaling pathway	0.008500	39	42
Bacterial invasion of epithelial cells	0.013550	54	51
Chronic myeloid leukemia	0.015818	52	53
Neurotrophin signaling pathway	0.015818	83	56
Tight junction	0.016385	94	54
T cell receptor signaling pathway	0.016385	71	54
Glycosaminoglycan biosynthesis—chondroitin sulfate/dermatan sulfate	0.022223	14	19
Protein processing in endoplasmic reticulum	0.022330	103	53
p53 signaling pathway	0.022460	48	49
SNARE interactions in vesicular transport	0.026510	25	37
cGMP-PKG signaling pathway	0.030693	107	53

## Data Availability

For preparation of the manuscript, the publicly available DIANA-microT web server v5.0 was used (http://diana.imis.athena-innovation.gr/DianaTools/index.php), accessed on 10 March 2022. miRWalk v 2.0 was used (http://mirwalk.umm.uni-heidelberg.de/), accessed on 12 March 2022. miRDB (http://mirdb.org.), accessed on 13 March 2022. TargetScanHuman v 8.0 was used (https://www.targetscan.org/vert_80/), accessed on 15 March 2022. miRTarBase v8.0 was used (https://mirtarbase.cuhk.edu.cn/~miRTarBase/miRTarBase_2022/php/index.php), accessed on 17 March 2022. Networks were created with the Cytoscape version 3.9.1. (https://cytoscape.org), accessed on 30 March 2022. miRPath v.3 was used for miRNA enrichment analysis (http://diana.imis.athena-innovation.gr/DianaTools/index.php), accessed on 15 April 2022. All the data are presented within the article.

## Supplementary Materials

The following supporting information can be downloaded at: https://www.mdpi.com/article/10.3390/genes13081443/s1, Supplement S1a–g: Network of predicted MTIs associated with PTH created with the Cytoscape software for miR-146a-5p, miR-551b-5p, miR-205-3p, miR-33a-3p, miR-338-5p and miR-410-3p. Orange-colored nodes represent miRNAs, while grey-colored nodes represent target genes. Red-colored edges represent validated interactions. The network contains 9676 nodes and 14,922 edges; Supplement S2: A heatmap of all significantly targeted pathways by PTH-regulated miRNAs.
