# Supplementary figures and images for: Interactome of PTH-Regulated miRNAs and Their Predicted Target Genes for Investigating the Epigenetic Effects of PTH (1–34) in Bone Metabolism
Source: Genes (Basel). 2022 Aug 13;13(8):1443. doi: 10.3390/genes13081443 (PMC9407897; doi:10.3390/genes13081443)

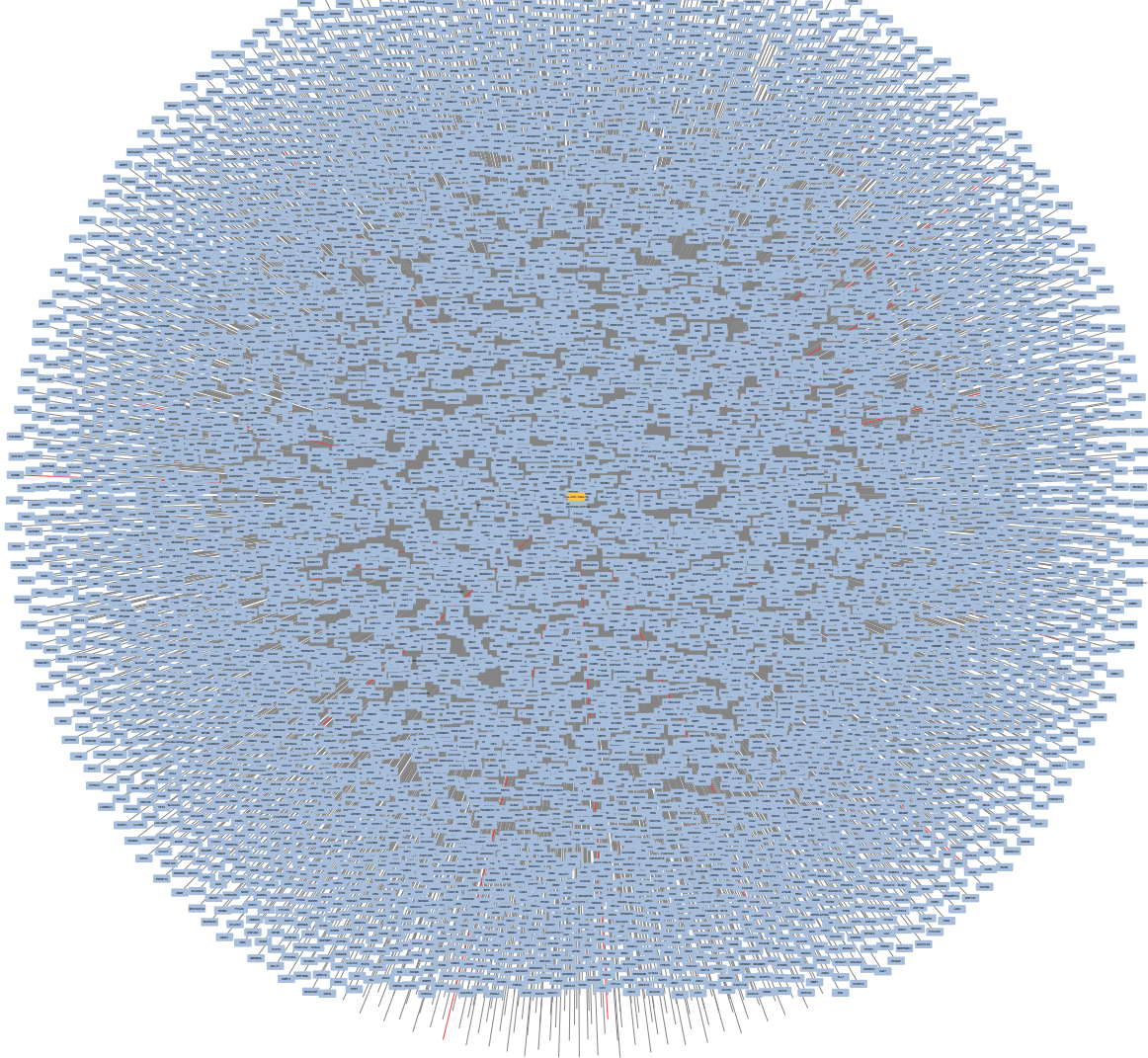

Supplement: Supplementary file 1 [file genes-13-01443-s001.zip › Genes-1838094 Suppl1/Suppl1b_Interactome of miR-146a-5p.pdf]

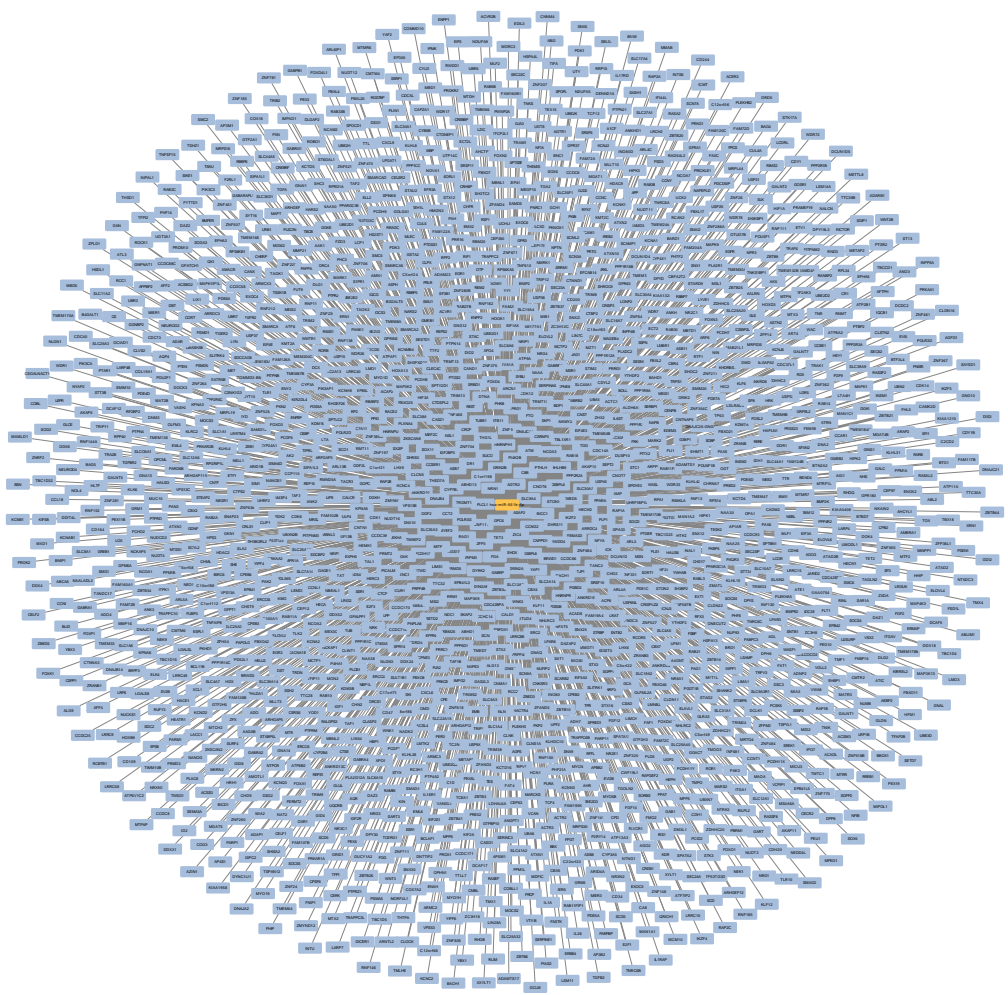

Supplement: Supplementary file 1 [file genes-13-01443-s001.zip › Genes-1838094 Suppl1/Suppl1c_Interactome of miR-551b-5p.pdf]

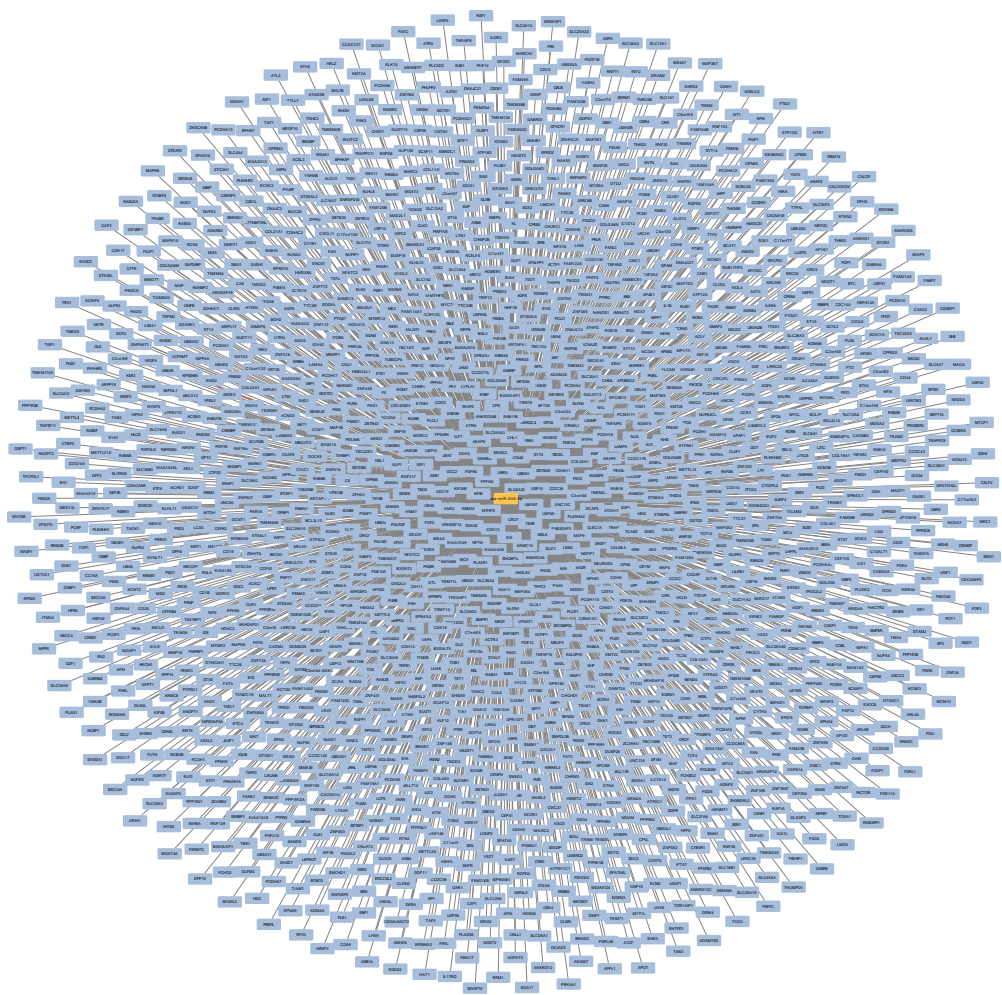

Supplement: Supplementary file 1 [file genes-13-01443-s001.zip › Genes-1838094 Suppl1/Suppl1d_Interactome of miR-205-3p.pdf]

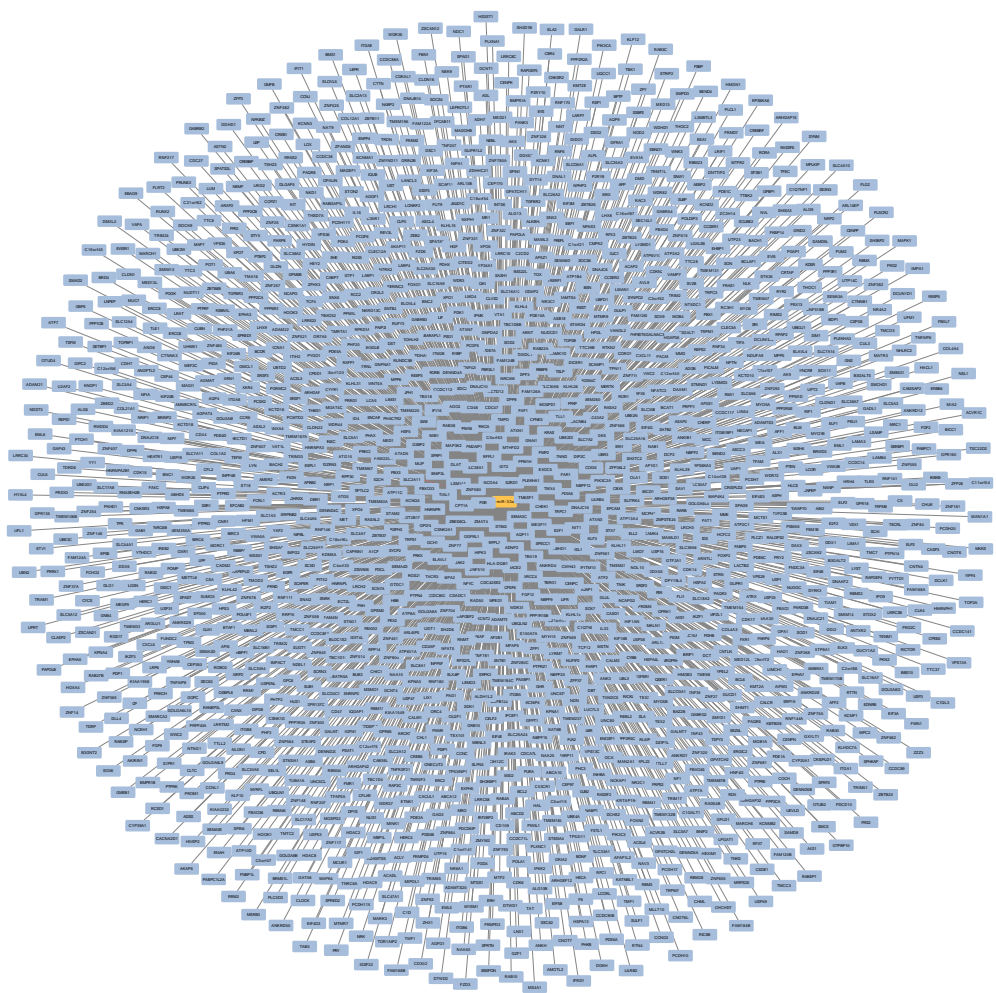

Supplement: Supplementary file 1 [file genes-13-01443-s001.zip › Genes-1838094 Suppl1/Suppl1e_Interactome of MiR-33a-3p.pdf]

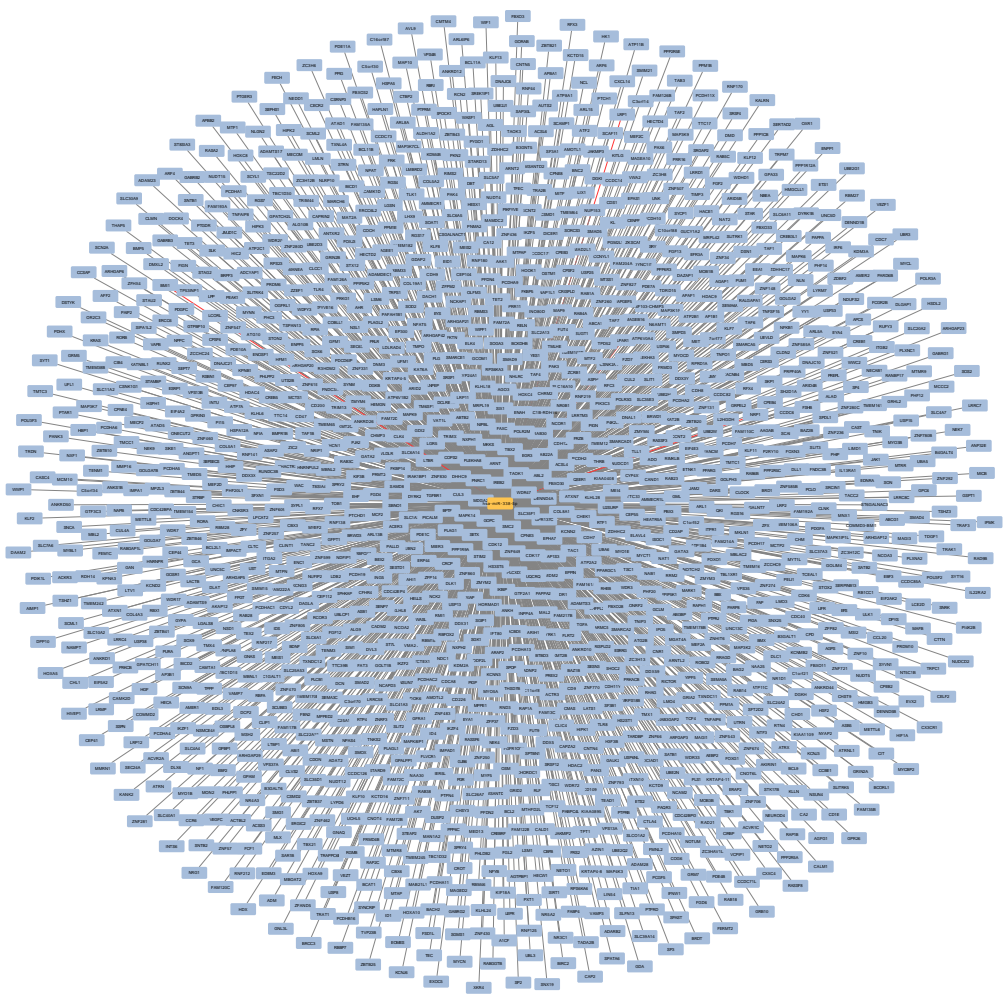

Supplement: Supplementary file 1 [file genes-13-01443-s001.zip › Genes-1838094 Suppl1/Suppl1f_Interactome of miR-338-5p.pdf]

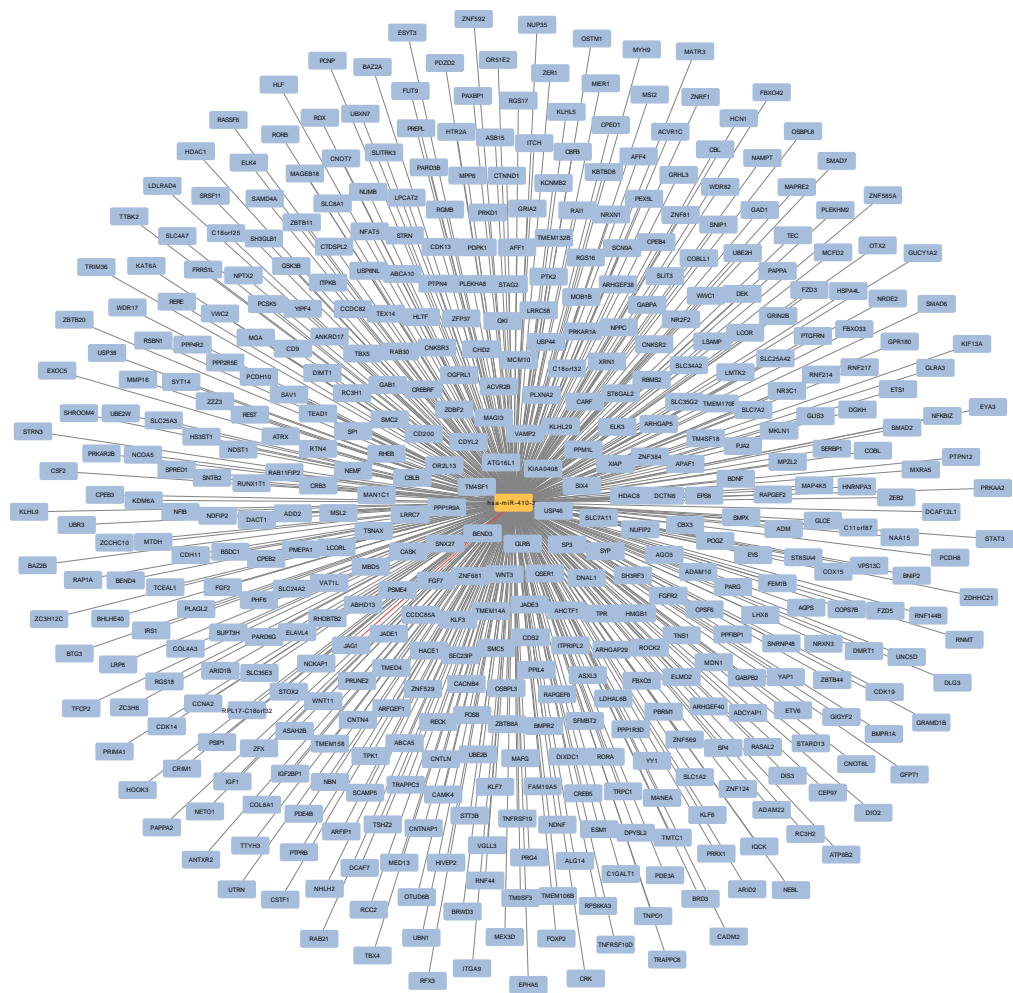

Supplement: Supplementary file 1 [file genes-13-01443-s001.zip › Genes-1838094 Suppl1/Suppl1g_Interactome of miR-410-3p.pdf]

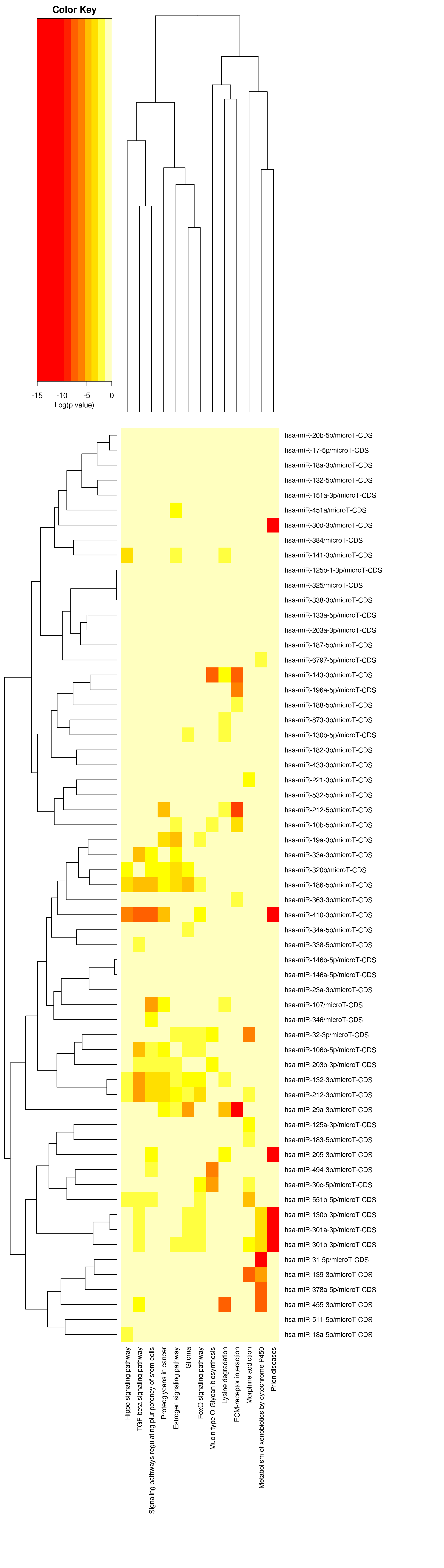

Supplement: Supplementary file 1 [file genes-13-01443-s001.zip › Genes-1838094 Suppl2/Heatmap of pathways union of 62 microRNAs.png.png]
